# Polarization and health-related behaviours and outcomes during the COVID-19 pandemic: a systematic review protocol

**DOI:** 10.12688/f1000research.145852.1

**Published:** 2024-05-17

**Authors:** Aziz Mert Ipekci, Maximilian Filsinger, Diana Buitrago-Garcia, Cristopher I. Kobler Betancourt, Annika Frahsa, Nicola Low

**Affiliations:** 1 Graduate School for Health Sciences, University of Bern, Bern, Switzerland; 2 Multidisciplinary Center for Infectious Diseases, University of Bern, Bern, Switzerland; 3 Institute of Social Preventive and Medicine, University of Bern, Bern, Switzerland; 4 Institute of Political Science, University of Bern, Bern, Switzerland

**Keywords:** Systematic review, political-polarization, affective-polarization, COVID-19, vaccination, social-distancing, infection-risk, mortality.

## Abstract

**Introduction:**

The COVID-19 pandemic affected people’s health behaviours and health outcomes. Political or affective polarization could be associated with health behaviours such as mask-wearing or vaccine uptake and with health outcomes, e.g., infection or mortality rate. Political polarization relates to divergence or spread of ideological beliefs and affective polarization is about dislike between people of different political groups, such as ideologies or parties. The objectives of this study are to investigate and synthesize evidence about associations between both forms of polarization and COVID-19 health behaviours and outcomes.

**Methods:**

In this systematic review, we will include quantitative studies that assess the relationship between political or affective polarization and COVID-19-related behaviours and outcomes, including adherence to mask mandates, vaccine uptake, infection and mortality rate. We will use a predetermined strategy to search EMBASE, Medline (Ovid), Cochrane Library, Cochrane COVID-19 Study Register, Global Health (Ovid), PsycInfo (Ovid), Web of Science, CINAHL, EconLit (EBSCOhost), WHO COVID-19 Database, iSearch COVID-19 Portfolio (NIH) and Google Scholar from 2019 to September 8 2023. One reviewer will screen unique records according to eligibility criteria. A second reviewer will verify the selection. Data extraction, using pre-piloted electronic forms, will follow a similar process. The risk of bias of the included studies will be assessed using the JBI checklist for analytical cross sectional studies. We will summarise the included studies descriptively and examine the heterogeneity between studies. Quantitative data pooling might not be feasible due to variations in measurement methods used to evaluate exposure, affective and political polarization. If there are enough relevant studies for statistical data synthesis, we will conduct a meta-analysis.

**Discussion:**

This review will help to better understand the concept of polarization in the context of the COVID-19 pandemic and might inform decision making for future pandemics.

**Protocol registration:**

PROSPERO ID: CRD42023475828.

## Introduction

During the COVID-19 pandemic, researchers observed variations in adherence to infection control measures, such as mask-wearing or vaccine uptake, ^1^^,^^2^ and in health outcomes, such as infection and mortality rate. ^3^ Political science research has long established that governmental action is often appraised through an ideological and partisan lens. ^4^^–^^6^ In this regard, increasing polarization of attitudes could contribute to explaining the variation in adherence to preventive behaviours and health outcomes. Studies have already shown that polarization of political or personal opinions can be associated with people’s COVID-19 pandemic behaviours and related health outcomes. ^7^^–^^9^ High levels of polarization might lead to poor health outcomes such as increased infection rate, reduced vaccine uptake or increased mortality ^7^^,^^8^^,^^10^^–^^12^ and lack of adherence to COVID-19 prevention measurements such as social distancing. ^13^

Affective and political polarization are related but different concepts. Political polarization refers to the degree to which political beliefs and opinions diverge along ideological lines, ^14^ whereas affective polarization refers to feelings of dislike and/or distrust that individuals or groups hold about those from a group with opposite views. ^15^ Political polarization can exist without affective polarization, which means people can have different political views without feeling hostile towards those with opposing views. Both political and affective polarization can be measured quantitatively ^16^^,^^17^ using tools based on self-report, such as the ideology scale, ^18^ feeling thermometer, ^19^ like-dislike ratings ^20^ and social distance scales. ^20^ Owing to differences between measurement methods, researchers should be cautious in comparing different measurement methods directly. ^18^

Within the research literature, a number of studies have focused on health-related behaviours and outcomes of polarization. Fraser and colleagues reported that in the United States of America (USA), based on polarization measured on a scale from 0 to 10, for each 1 unit increase in state-level perceived polarization the incidence rate of experiencing poor physical health increased by 1.03 times. ^21^ Krupenkin studied the effects of political partisanship on children’s vaccination rate. They dichotomised people into in-partisans (people who voted for the government in power), and out-partisans (people who voted against the government in power). ^8^ In a multivariable logistic regression model, presidential out-partisans had lower odds of adhering to USA Government vaccination recommendation than in-partisans. ^8^ Nayak and colleagues measured both perceived polarization change and self-reported health with a 5-point Likert scale. ^22^ They found that individuals who reported higher levels of polarization had higher odds of developing depressive and anxiety disorders than those who reported no change in polarization. ^22^ In the context of the COVID-19 pandemic, Gollwitzer et al. studied partisanship at the county level in the USA based on the 2016 presidential election and reported that pro-Trump counties reduced their general movement 9.5 per cent less than Clinton-voting counties. ^7^

We found two systematic reviews on polarization but they focus on the association with social media. ^18^^,^^23^ Both conducted descriptive syntheses of the data, ^18^^,^^23^ with Kubin and colleagues stating that they were unable to perform meta-analysis due to inconsistencies in measurement. ^18^ To our knowledge, there are no systematic reviews focusing on the association between polarization and health-related health behaviours or outcomes despite the consistent associations found between different forms of polarization and health-related behaviours. This systematic review aims to fill the gap in the literature on the association between polarization and COVID-19 related health behaviours/outcomes to better understand the COVID-19 pandemic and prepare for future pandemics.

## Review questions

Question 1: What is the association between political or affective polarization and COVID-19 health behaviours?

Question 2: What is the association between political/affective polarization and COVID-19-related health outcomes?

## Methods

This protocol is reported following the Systematic Reviews and Meta-Analysis Protocols (PRISMA-P) guideline (Extended data A), ^24^ PROSPERO registration number, CRD42023475828.

### Eligibility criteria

**Table:** 

Inclusion criteria	Study population: individuals of any age and gender. Exposure: Affective and political polarization measured quantitively Outcome: COVID-19 infection risk, COVID-19 hospitalization risk, COVID-19 mortality risk, COVID-19 vaccine uptake, compliance with mask wearing advice, compliance with physical distancing advice, perceived COVID-19 risk. Publication type: Manuscript reporting primary data, irrespective of publication status. No language restriction.
Eligible study designs	Cohort studies Case-control studies Cross-sectional studies Ecological studies
Exclusion criteria	No additional exclusion criteria
Excluded study designs	Reviews, editorials or commentaries not reporting original data

### Search strategy

We searched electronic databases using predefined terms for polarization and COVID-19 (Extended data B) on 8th of September 2023. We will include studies published from 2019 to 2023. Because the topic is multidisciplinary, we will search the following databases: EMBASE (RRID:SCR_001650), Medline (Ovid), Cochrane Library (RRID:SCR_013000), Cochrane COVID-19 Study Register, Global Health (Ovid), PsycInfo (Ovid), Web of Science (RRID:SCR_022706, CINAHL (RRID:SCR_022707), EconLit (EBSCOhost), We will use WHO COVID-19 Database and iSearch COVID-19 Portfolio (NIH) (RRID:SCR_018295) as the source of preprint publications. We will also run a Google Scholar (RRID:SCR_008878) search using keywords such as polarization, affective, political and COVID-19. We will review the first 200 hits on Google Scholar to see if we can identify any study that cannot be identified via our literature search. We will check the reference lists of relevant studies and systematic reviews. We will also contact experts in the field to ask for recommendations about studies that might be eligible. We will not perform hand-searching. We will merge the electronic database search results and remove duplicates using reference management software (EndNote – Clarivate, version 20.4).

### Screening and study selection

We will use the liberal screening approach ^25^ to accelerate our screening process. AMI will screen all titles and abstracts and select potentially relevant articles according to the eligibility criteria. A second reviewer (MF, AF, CK-B or DB-G) will verify the screened articles. AMI will retrieve the full-text of all potentially eligible articles and mark those eligible for inclusion. MF, AF, CK-B or DB-G will verify the results of the full-text screening. In case of disagreements that are not resolved by discussion, the senior reviewer NL will decide. We will report the study selection process, and reasons for exclusion, in the PRISMA 2020 ^26^ flow diagram.

### Data extraction

We will use a predetermined data extraction form in the Covidence systematic review software (Veritas Health Innovation, Melbourne, Australia, available at www.covidence.org, RRID:SCR_016484). We piloted extraction from 5 included studies. We will revise and finalize the form (Extended data C) after our pilot extraction. We plan to extract data on how polarization and COVID-19 related health behaviours/outcomes were measured, the main findings, and possible confounding factors, such as data collection date, participant’s age, gender and socioeconomic status. The full list of questions can be found in Extended data C. AMI will extract data from all included articles and MF, AF, CK-B and DB-G will independently verify the accuracy of the extracted data. NL will resolve disagreements if necessary.

### Dealing with missing data

We will contact corresponding authors in case of any missing data in the included study. If the author does not reply, researchers (AMI, MF) will decide on whether the study can still be included.

### Quality and risk of bias assessment

AMI, MF and DB-G will assess risk of bias independently for each included study. NL will resolve disagreements if the two reviewers cannot reach a consensus. We will use the JBI checklist for analytical cross sectional studies. ^27^

### Data synthesis and analysis

The data analysis will start with a description of countries of origin, study population, the methods used to measure exposure and outcome, and the participants’ age and sex in the included studies.

We will employ narrative synthesis methods to explore our dataset following the Synthesis Without Meta-analysis guideline. ^28^ We will group the studies for synthesis based on exposure, affective or political polarization, and outcome, e.g., vaccination uptake and perceived COVID-19 risk. Then, we will describe the metrics for each exposure and outcome. We will justify our reasoning, if certain studies are prioritized to draw conclusions. Lastly, we will report on the heterogeneity and assess the certainty of the synthesis findings.

Our preliminary overview of the literature indicated that there might be too few comparable studies for quantitative data synthesis, owing to variations in measurement methods used to evaluate exposure, and affective and political polarization. Additionally, the potential for heterogeneity exists due to differences in study setups, countries of origin and pandemic severity at the time of study data collection.

We will examine statistical heterogeneity using the I-squared statistic if there are estimated proportions from three or more studies. ^29^ After considering sources of heterogeneity, we will decide if statistically combining effect estimates with a meta-analysis is appropriate for included studies. ^30^

### Dissemination

The results of this study will be published in a peer-reviewed journal.

### Study status

The literature search for the study has been done. Screening is ongoing, the data extraction, risk of bias analysis, data synthesis and writing of the final report have not started yet.

## Discussion

Our study has two main strengths. First, our comprehensive search strategy includes both preprint and published articles gathered from a range of databases in health and political sciences. This will ensure the incorporation of evidence from various fields. Second, our team includes experts with varied backgrounds, including epidemiology, medicine, political sciences, and anthropology, ensuring a wide range of perspectives. This diverse outlook will enable us to adopt a comprehensive approach to both analysis and data interpretation.

Our review also has weaknesses. We will not perform independent screening and extraction in our systematic review owing to time and resource constraints. However, the liberal approach, to include more articles for full-text screening, will reduce the risk of missing important articles. Second, it might not be possible to pool the data quantitatively. Narrative synthesis methods will, however, provide a valid interpretation of the data.

Our preliminary search shows a need for a systematic literature review and evidence synthesis on the association between pandemic related health behaviours/outcomes and polarization. Our systematic review aims to fill the gap in the literature to better understand the COVID-19 pandemic, which could inform decision making for future pandemics.

### Ethic and consent

Ethical approval and written consent were not required.

## Data availability

### Underlying data

No data is associated with this article.

### Extended data

OSF: Extended Data, https://doi.org/10.17605/OSF.IO/DG87Q. ^31^

This project contains the following underlying data:

A. PRISMA-P (Preferred Reporting Items for Systematic review and Meta-Analysis Protocols) 2015 checklist: recommended items to address in a systematic review protocol
B. Full search strategy per database
C. Data extraction form.

The data are available under the terms of the Creative Commons Attribution 4.0 International license (CC-BY 4.0).

### Reporting guidelines

OSF: Checklist for Polarization and health-related behaviours and outcomes during the COVID-19 pandemic: a systematic review protocol, https://doi.org/10.17605/OSF.IO/DG87Q. ^31^

## Software availability

Covidence (Veritas Health Innovation, Melbourne, Australia, available at www.covidence.org). is a proprietary software. An alternative software that can be used for free is Rayyan ( https://www.rayyan.ai/) that allows management and organization of systematic reviews.
