# Supplementary material for: Polarization and health-related behaviours and outcomes during the COVID-19 pandemic: a systematic review protocol
Source: F1000Res. 2024 May 17;13:488. [Version 1] doi: 10.12688/f1000research.145852.1 (PMC11255549; doi:10.12688/f1000research.145852.1)
Supplement: Peer review history

# Reviewer report: Reviewer response for version 1

**DOI:** 10.5256/f1000research.159858.r298434

**Published:** 2024-07-17

**Authors:** Markus Wagner

**Affiliations:** 1 University of Vienna, Vienna, Austria

This systematic review protocal addresses an important and relevant question concerning health behaviours and health outcomes, namely the impact of various types of political polarization. Given what we know from political science, it is highly likely that political ideologies and partisanship will influence how people behave, and people may even take decisions that will harm themselves due to their political biases.

 This protocol is clear and transparent, and all key steps are described well. All procedures conform to best practice, at least to my knowledge. I have a couple of comments that may be useful to the researchers:

 - The protocal addresses political polarization and affective polarization. I would suggest a different way of capturing this, as I think most political scientists would argue that there is an overarching phenomenon of political polarization, which can be subdivided into ideological polarization and affective polarization. The former is what the authors here call "political" polarization, but I think that is a little misleading, as affective polarization is also political. 

 - Within affective polarization, it would be important to pay additional attention to the role of partisanship and party identities more explicitly. This could encompass both positive and negative partisan identities.

 - An important additional aspect of interest could be to examine whether polarization from/by the left or from/by the right has more of an impact on health behaviours and outcomes. Polarization is a deceptively neutral term, and may be the result of growing extremism and stronger affective patterns on both sides of the political spectrum. Including this in the analysis as best possible would be an important addition.

Is the study design appropriate for the research question?

Yes

Is the rationale for, and objectives of, the study clearly described?

Yes

Are sufficient details of the methods provided to allow replication by others?

Yes

Are the datasets clearly presented in a useable and accessible format?

Yes

Reviewer Expertise:

Political science

I confirm that I have read this submission and believe that I have an appropriate level of expertise to confirm that it is of an acceptable scientific standard.

# Reviewer report: Reviewer response for version 1

**DOI:** 10.5256/f1000research.159858.r287860

**Published:** 2024-06-19

**Authors:** Ray Block

**Affiliations:** 1 The Pennsylvania State University - University Park Campus, University Park, Pennsylvania, USA

I am pleased to support the advancement of the manuscript titled "Polarization and Health-Related Behaviours and Outcomes During the COVID-19 Pandemic: A Systematic Review Protocol."

 The authors are addressing a crucial aspect of the pandemic by synthesizing the existing (quantitative) empirical literature on polarization and COVID-19 mitigation behaviors. This work is not only timely but also essential in understanding how societal divisions affect health-related behaviors and outcomes.

 One of the standout features of this manuscript is its ambitious scope. The authors aim to synthesize research from various geographic contexts over multiple years of the pandemic. Additionally, they consider a broad range of mitigation behaviors, such as masking and vaccine uptake, and examine different forms of polarization, including affective and political. This comprehensive approach is commendable and will provide valuable insights into the complex interplay between polarization and health behaviors during the COVID-19 pandemic.

 The research design outlined by the authors is detailed and appropriate for a literature synthesis of this magnitude. While there may be concerns about the availability of a sufficient number of studies to conduct a statistical analysis of effect sizes, I am hopeful that such meta-analyses will be feasible. Having this information could significantly inform future policymaking, both for the ongoing pandemic and for future public health emergencies.

 The author team is uniquely positioned to undertake and complete this ambitious project. I look forward to seeing future iterations and the final outcomes of this research. Their work will undoubtedly contribute significantly to the understanding of polarization and health-related behaviors during the COVID-19 pandemic.

 I have minimal critique to offer this study. My main suggestion is the inclusion of research conducted by my colleagues, such as [1],[2]. These studies could add valuable data to the synthesis.

Is the study design appropriate for the research question?

Yes

Is the rationale for, and objectives of, the study clearly described?

Yes

Are sufficient details of the methods provided to allow replication by others?

Yes

Are the datasets clearly presented in a useable and accessible format?

Yes

Reviewer Expertise:

Political science (race and ethnic politics, political behavior, public opinion)

I confirm that I have read this submission and believe that I have an appropriate level of expertise to confirm that it is of an acceptable scientific standard.

## References

[1] : The Self-Appraisal of Masking Instrument. *Measurement Instruments for the Social Sciences* .2022;4(1) : 10.1186/s42409-022-00032-3 10.1186/s42409-022-00032-3

[2] : Perceived risk, political polarization, and the willingness to follow COVID-19 mitigation guidelines. *Soc Sci Med* .2022;305: 10.1016/j.socscimed.2022.115091 115091 10.1016/j.socscimed.2022.115091 35690035 PMC9161674
